# Whole-Brain Imaging of Subvoxel T1-Diffusion Correlation Spectra in Human Subjects

**DOI:** 10.3389/fnins.2021.671465

**Published:** 2021-06-11

**Authors:** Alexandru V. Avram, Joelle E. Sarlls, Peter J. Basser

**Affiliations:** ^1^Eunice Kennedy Shriver National Institute of Child Health and Human Development, National Institutes of Health, Bethesda, MD, United States; ^2^Center for Neuroscience and Regenerative Medicine, The Henry M. Jackson Foundation for the Advancement of Military Medicine, Bethesda, MD, United States; ^3^National Institute of Neurological Disorders and Stroke, National Institutes of Health, Bethesda, MD, United States

**Keywords:** isotropic diffusion encoding, T1-diffusion weighting, correlation spectroscopic MRI, multidimensional MRI, relaxation spectroscopy MRI, mean diffusivity distribution, relaxographic imaging, inversion recovery

## Abstract

T1 relaxation and water mobility generate eloquent MRI tissue contrasts with great diagnostic value in many neuroradiological applications. However, conventional methods do not adequately quantify the microscopic heterogeneity of these important biophysical properties within a voxel, and therefore have limited biological specificity. We describe a new correlation spectroscopic (CS) MRI method for measuring how T1 and mean diffusivity (MD) co-vary in microscopic tissue environments. We develop a clinical pulse sequence that combines inversion recovery (IR) with single-shot isotropic diffusion encoding (IDE) to efficiently acquire whole-brain MRIs with a wide range of joint T1-MD weightings. Unlike conventional diffusion encoding, the IDE preparation ensures that all subvoxel water pools are weighted by their MDs regardless of the sizes, shapes, and orientations of their corresponding microscopic diffusion tensors. Accordingly, IR-IDE measurements are well-suited for model-free, quantitative spectroscopic analysis of microscopic water pools. Using numerical simulations, phantom experiments, and data from healthy volunteers we demonstrate how IR-IDE MRIs can be processed to reconstruct maps of two-dimensional joint probability density functions, i.e., correlation spectra, of subvoxel T1-MD values. *In vivo* T1-MD spectra show distinct cerebrospinal fluid and parenchymal tissue components specific to white matter, cortical gray matter, basal ganglia, and myelinated fiber pathways, suggesting the potential for improved biological specificity. The one-dimensional marginal distributions derived from the T1-MD correlation spectra agree well with results from other relaxation spectroscopic and quantitative MRI studies, validating the T1-MD contrast encoding and the spectral reconstruction. Mapping subvoxel T1-diffusion correlations in patient populations may provide a more nuanced, comprehensive, sensitive, and specific neuroradiological assessment of the non-specific changes seen on fluid-attenuated inversion recovery (FLAIR) and diffusion-weighted MRIs (DWIs) in cancer, ischemic stroke, or brain injury.

## 1. Introduction

T1-weighted (T1W) MRIs, such as fluid-attenuated inversion recovery (FLAIR) (Bydder and Young, 1985; De Coene et al., 1992; Hajnal et al., 1992) or MP-RAGE (Mugler and Brookeman, 1990) images reflect differences in average T1 relaxation times of tissues. Meanwhile, diffusion-weighted MRIs (DWIs) reflect differences in average tissue water diffusion properties (Bihan et al., 1986; Moseley et al., 1990), such as the mean apparent diffusion coefficients (mADCs), or the mean diffusivities (MDs) (Basser et al., 1994). Both T1- and diffusion-weighted MRIs provide excellent tissue contrasts and are indispensable in many neurological and neuroradiological applications. These two contrasts are almost always acquired in separate scans, and often yield complementary radiological information crucial to the study of neurodegenerative diseases (Werring et al., 1999; Schmidt et al., 2012), cancer (Brunberg et al., 1995; Essig et al., 1998), ischemic stroke (Lutsep et al., 1997; Kamran et al., 2000), neuroinflammation (Ashikaga et al., 1997; McKinstry et al., 2002), traumatic brain injury (TBI) (Shenton et al., 2012), and brain development (Neil et al., 1998; Ashikaga et al., 1999). The enduring utility of T1W and DWIs in radiological sciences is strong evidence of the high sensitivity of T1 and diffusion to a wide range of pathophysiological processes and motivates efforts to advance the quantitative mapping of these important biophysical tissue properties.

Despite their widespread use, however, conventional weighted MRIs do not directly quantify the underlying T1 and diffusion tissue properties. Therefore, signal changes seen during disease cannot be traced back to alterations in specific tissue components. Quantitative MRI (qMRI) explicitly accounts for the most important experimental factors (Helms et al., 2011; Weiskopf et al., 2013), e.g., pulse sequence, TE, TR, *b*-value, to measure voxel-averaged values of tissue T1 and MD. Nonetheless, these voxel-averaged estimates produce a mere phenomenological description of the tissue composition, assuming homogeneity within the imaging voxel. Clearly, there is a critical need to quantify the microscopic heterogeneity of these parameters in healthy and diseased brain tissues using a model-free, non-parametric approach (Avram et al., 2019).

Relaxation-spectroscopy (RS) MRI, or relaxographic imaging (Labadie et al., 1994), combines NMR relaxometry (Kroeker and Mark Henkelman, 1986; English et al., 1991; Does and Snyder, 1995; Does et al., 1998; Ronen et al., 2006) with MR imaging to quantify the subvoxel (i.e., microscopic) heterogeneity of parameters such as T1, T2, or diffusivity from multiple images acquired with different contrast weightings. For example, from MRIs measured with multiple TEs one can derive the distribution (or spectrum) of subvoxel T2 values and compute maps of myelin water fraction, defined as the short-T2 signal component in white matter (WM) (Mackay et al., 1994; Whittall et al., 1997). Despite the early success of imaging T2 spectra in the human brain, the mapping of T1 spectra (Labadie et al., 2014) *in vivo* proved technically challenging due to factors such as long scan durations or imperfect adiabatic inversion. Nevertheless, a few clever techniques were developed to directly image short-T1 components in WM, believed to be associated with myelin water (Deoni et al., 2008; Oh et al., 2013).

Meanwhile, the clinical mapping of diffusivity spectra in tissues poses a unique challenge due to the need to account for diffusion anisotropy. Using conventional, single diffusion encoding (Stejskal and Tanner, 1965) it is possible to measure spectra of diffusivities along a given orientation. The mADC-weighted signal obtained by averaging DWIs acquired with diffusion gradients applied along uniformly distributed orientations (Jones et al., 1999) at a fixed b-value removes signal variations due to diffusion anisotropy that manifest at the macroscopic (voxel) scale. These signal variations (Avram et al., 2018) can be described phenomenologically using methods such as diffusion tensor imaging (DTI) (Basser et al., 1994), which view tissues as isotropic or anisotropic media that are homogeneous at the microscopic scale. In homogeneous media, the voxel-averaged (macroscopic) diffusion tensor measured with DTI also describes diffusion at the microscopic scale. Hence, the MD measured as one-third of the Trace of the diffusion tensor reflects an intrinsic property of the medium, and is equal to the mADC. In neural tissues, however, the heterogeneous microstructure gives rise to diverse diffusion processes in subvoxel water pools. These processes can be described with distinct diffusion tensors of various sizes (mean diffusivities), shapes (anisotropies), and orientations (principal diffusion directions). Measurements acquired with single diffusion encoding used to derive mADC-weighted signals are not sensitive to the correlations between the properties of these tensors (sizes, shapes, and orientations). Therefore, the mADC-weighted signals do not properly remove specific effects from only the orientation dispersion or diffusion anisotropies at the microscopic scale.

On the other hand, measurements acquired with multiple diffusion encoding (Mitra, 1995; Callaghan and Komlosh, 2002; Westin et al., 2016; Topgaard, 2017) are sensitive to diffusion-diffusion correlations, allowing us to probe the tensor characteristics (size, shape, orientation) of diffusion processes in microscopic water pools and to measure microscopic diffusion anisotropy (Avram et al., 2013) in healthy subjects and patients (Szczepankiewicz et al., 2015, 2016). Moreover, isotropic diffusion encoding (IDE) (Avram et al., 2019), or spherical tensor encoding (Eriksson et al., 2013; Westin et al., 2016; Topgaard, 2017), directly sensitizes the signals from all subvoxel water pools to their intrinsic MDs, regardless of their microscopic anisotropies and preferred diffusion orientations (Avram et al., 2019). Since IDE-prepared measurements are superpositions of signals from all subvoxel water pools, each weighted by its corresponding MD, they can be analyzed with RS-MRI to retrieve the subvoxel distribution of intrinsic MD values. This very efficient, practical, and clinically feasible method provides whole-brain maps of subvoxel MD spectra in only 6 min (Avram et al., 2019).

Quantifying correlations between multiple relaxation mechanisms, e.g., T1, T2, diffusion, may further improve biological specificity. From relaxation correlation spectroscopy NMR measurements acquired by varying the joint weighting (e.g., TE and *b*-value) of multiple contrasts (e.g., T2 and diffusivity, respectively) one can derive a multidimensional spectrum of the corresponding biophysical parameters. Such experiments have been conducted in porous media (Hürlimann et al., 2002, 2003; de Almeida Martins and Topgaard, 2018), biological samples (English et al., 1991; Does and Gore, 2002; Travis and Does, 2005), and human volunteers (Saab et al., 2001). The imaging extension of this spectroscopic NMR method, multidimensional correlation spectroscopic (CS) MRI provides a correlation spectrum in each voxel. Microimaging (Benjamini and Basser, 2017; Kim et al., 2017; Yon et al., 2020) and preliminary *in vivo* CS-MRI studies (Tax et al., 2017; Hutter et al., 2018; Kim et al., 2018, 2020; Slator et al., 2019) have focused on combining T2 (or T2^*^) with T1 (Kim et al., 2018) or conventional diffusion encoding (primarily in excised samples with well-oriented microstructure, such as the spinal cord) and have shown improvements in specificity (Benjamini and Basser, 2017; Kim et al., 2017).

In this study, we develop a new method for mapping two-dimensional probability density functions (or spectra) of subvoxel T1-MD values in human subjects, thereby addressing a critical step in the clinical translation of multidimensional CS-MRI. Specifically, we design and evaluate a pulse sequence that integrates inversion recovery (IR) and IDE (Avram et al., 2019) preparations to measure for the first time whole-brain MRIs with a wide range of joint T1-MD contrasts using a conventional clinical scanner. We describe how IR-IDE MRIs with multiple weightings can be processed to derive T1-MD correlation spectra in brain tissues. The non-invasive, model-free, and whole-brain assessment of T1-MD correlations revealing distinct microscopic water pools could provide a more nuanced, quantitative, and specific diagnosis of many pathophysiological processes.

## 2. Methods

### 2.1. IR-Prepared IDE MRI Pulse Sequence

**Figure 1:**
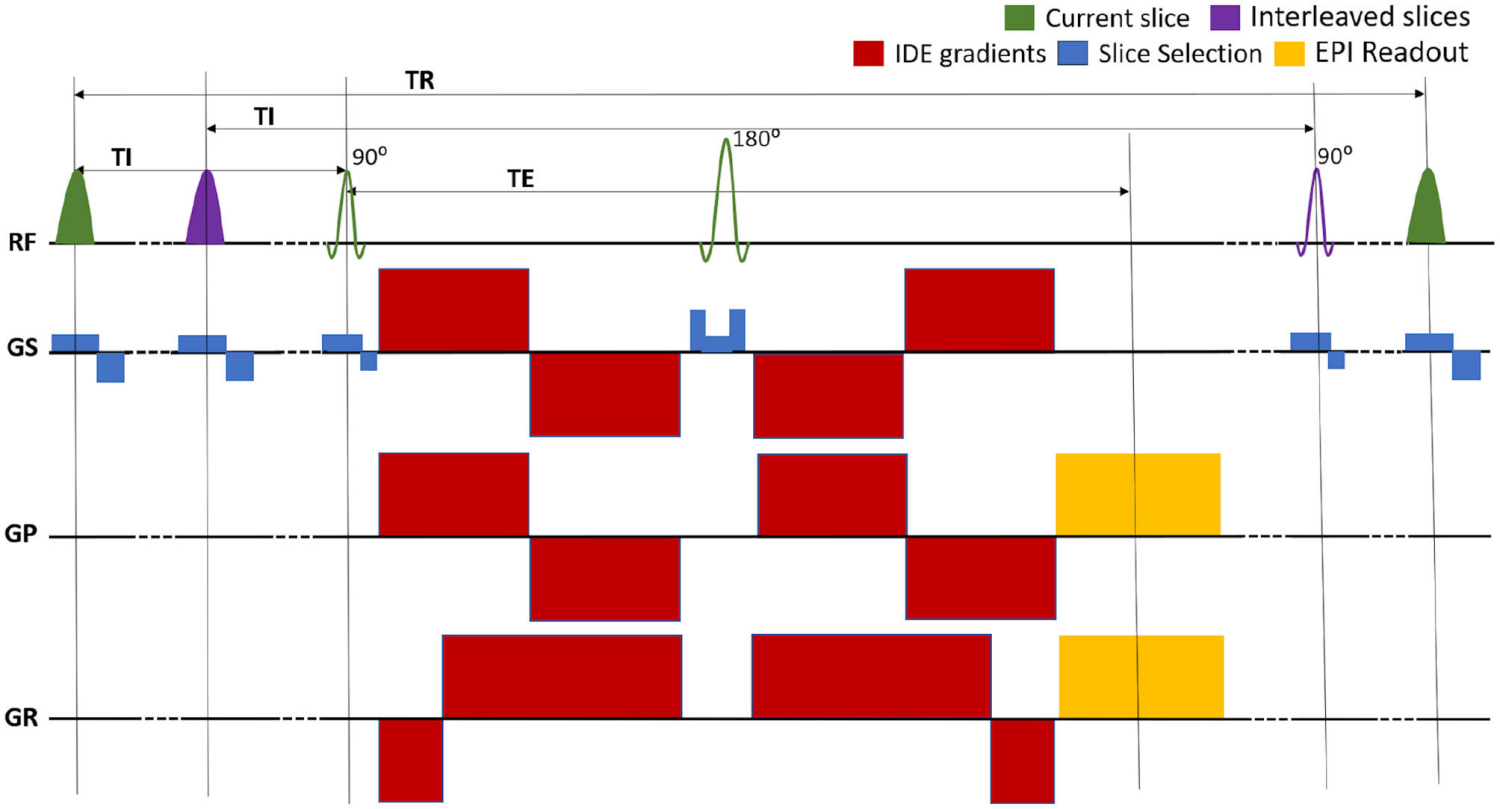
Pulse sequence diagram for the multislice EPI acquisition of MRIs with integrated inversion recovery (IR) and isotropic diffusion encoding (IDE) preparations. The slice-selective IR preparations and EPI acquisitions are interleaved so as to maintain the same inversion time (TI) and repetition time (TR) for each slice. The TR can be adjusted to minimize the total scan time. The experiment consists of separate repeated IR-IDE scans with different T1-weightings determined by fixing TI and TR. For each scan, IDE-DWIs with different b-values are acquired in consecutive TRs. The TE is fixed for all measurements.

We implemented a repeated IR spin-echo EPI pulse sequence with IDE preparation that allows independent control of T1 and MD weightings (Figure 1). The sequence is used to acquire whole-brain DWIs with a conventional single-shot EPI readout trajectory to prevent potential imaging artifacts arising from motion-induced phase inconsistencies in multi-shot *in vivo* acquisitions of diffusion-prepared signals.

The IR-preparation consists of a slice-selective adiabatic radio-frequency (RF) inversion pulse with a hyperbolic secant amplitude envelope and a 10.2 ms duration, followed by a gradient crusher. The IR-preparation and corresponding slice acquisition modules are interleaved to maintain the same TI and TR values for all slices (Park et al., 1985; Oh et al., 1991; Listerud et al., 1996). For any given slice, the slice-selective IR RF pulse excites the same location as the corresponding excitation and refocusing RF pulses but uses a 1.25 larger slice thickness to mitigate the loss of inversion efficiency due to mismatch of slice profiles. Moreover, to prevent cross-talk between IR pulses in adjacent slices, even and odd slices can be acquired in separate packets, with interleaved slice acquisition orders within each packet. The duration between two consecutive IR modules (and slice acquisitions) is determined by the TR and the number of slices per packet. In each scan, the T1-weighting is determined by fixing both TI and TR, while in consecutive TRs the diffusion-weighting is adjusted to acquire IR-DWIs with different *b*-values. The complete experiment consists of several repeated IR-prepared EPI scans, each with a different set of fixed (TI, TR) parameters determining different T1-weightings. To account for T1-encoding, both TI and TR are explicitly incorporated into the signal equation, as described in the following section.

The IDE-preparation module consists of a numerically optimized diffusion-weighting gradient waveform (Sjölund et al., 2015; Avram et al., 2019) that provides isotropic diffusion encoding in a single excitation (Mori and Van Zijl, 1995; Wong et al., 1995), but with a more efficient allocation of clinical diffusion gradient pulses before and after the 180° RF pulse (Avram et al., 2019). The desired maximum *b*-value determines the timings of the diffusion gradient pulses, and consequently the minimum TE. Rotation-invariant measurements with different *b*-values are acquired by simply scaling down the diffusion gradient pulse amplitudes. If we assume that the voxel is composed of multiple water pools, each with its own microscopic diffusion tensor whose corresponding diffusion ellipsoid has an arbitrary size, shape, and orientation, then the IDE preparation provides isotropic weighting by the Trace, or MD, for each of these microscopic tensors (Avram et al., 2019), allowing a spectroscopic quantitation of subvoxel MDs. Images with a fixed T1-weighting (TI and TR) but different *b*-values can be acquired efficiently in consecutive TRs of the same repeated IR scan. Because the T1 and diffusion weightings are encoded in the longitudinal and transverse magnetizations respectively, we can acquire signals with an arbitrary range of joint TI-b parameters. This contrasts with T2-diffusion CS-MRI measurements, where the practical range of joint TE-b parameters is limited by the minimum achievable TE as a function of *b*-value.

For a repeated IR spin-echo experiment (Figure 1) with perfect adiabatic inversion, the signal attenuation due to T1 and diffusion weightings in a homogeneous spin system with longitudinal relaxation time constant *T*_1_ and mean diffusivity $\bar{D}$ is:

(1) $$\begin{array}{l} S\left(b,TI,TR\right)=\left(1-2e^{-\frac{TI}{T_{1}}}+e^{-\frac{TR}{T_{1}}}\right)e^{-b\cdot \bar{D}} \end{array}$$

By systematically varying the weighting parameters we can efficiently acquire multiple magnitude IR-IDE DWIs with a wide range of combined T1-MD weightings.

### 2.2. Polarity Estimation of IR-IDE MRIs

Subject and physiological motions can produce undesired phase contributions in DWIs acquired *in vivo*. Therefore, we can only reliably measure magnitude IR-IDE DWIs. However, in order to discriminate a superposition of longitudinal inversion recovery processes from subvoxel water pools with distinct T1s it is necessary to use phase-sensitive IR data that can capture both positive and negative values of longitudinal magnetization within a voxel. We adapted a simple method (Bakker et al., 1984) for estimating the polarity of IR measurements from multiple measurements with different TIs. Specifically, for a series of magnitude measurements |**M**(*TI*_*k*_)|, associated with the increasing sequence of inversion times, *TI*_*k*_, we first identify the minimum magnitude signal |*M*_*m*_| = min|**M**(*TI*_*k*_)| and its corresponding *TI*_*m*_. Next, we invert the polarity of all magnitude measurements |*M*_*k*_| with *TI*_*k*_ < *TI*_*m*_ and remove the minimum point at *TI*_*m*_, to create a set of phase-sensitive IR data points. Finally, we interpolate the value at *TI*_*m*_, compare it to both |*M*_*m*_| and − |*M*_*m*_| and select the polarity of *M*_*m*_ as the closest of the two points to the interpolated value at *TI*_*m*_.

### 2.3. Estimating T1-MD Relaxation Correlation Spectra

In a voxel containing tissue with heterogeneous T1-MD properties, the net signal can be described as a superposition of contributions from microscopic water pools with arbitrary T1 and MD properties. Assuming slow exchange between these subvoxel components, we can write the net signal attenuation as a function of the contrast weighting parameters b, TI, and TR:

(2) $$\begin{array}{l} S\left(b,TI,TR\right)=\int_{0}^{\infty }\int_{0}^{\infty }(1-2e^{-TI\cdot R_{1}}\\ \;+e^{-TR\cdot R_{1}})e^{-b\cdot \bar{D}}p(R_{1},\bar{D})dR_{1}d\bar{D} \end{array}$$

where, *R*_1_ = 1/*T*_1_ and $\bar{D}$ are the longitudinal magnetic relaxation rate and mean diffusivity, respectively, and $p\left(R_{1},\bar{D}\right)$ is the two-dimensional joint probability density function of *R*_1_ and $\bar{D}$ values within the voxel. A piecewise continuous approximation of $p\left(R_{1},\bar{D}\right)$ can be estimated numerically by solving the linear system **M** · **p** = **y**, with **y** and **p** representing column vectors containing the measured IR-IDE signal attenuations and the unknown signal fractions of T1-MD spectral components, respectively. The elements of the encoding matrix **M**, *M*_*ij*_, are computed as the mean signal attenuations in the i-th experiment, *S*(*b*_*i*_, *TI*_*i*_, *TR*_*i*_), evaluated over the 2D box function determined by the intervals [*R*_1,*j*_, *R*_1,*j*+1_] and $\left[{\bar{D}}_{j},{\bar{D}}_{j+1}\right]$ corresponding to the j-th spectral bin in the piecewise continuous approximation of $p\left(R_{1},\bar{D}\right)$.

Because this problem is poorly conditioned, additional regularization is required to obtain a stable solution. A straightforward approach is to solve the problem by least-squares minimization using L2-norm regularization and positivity constraints:

(3) $$\begin{array}{l} \hat{\text{p}}=\underset{\text{p}>0}{\text{argmin}}{\left|\left|\left[\begin{array}{c} \text{M}\\ \lambda \text{I} \end{array}\right]\text{p}-\left[\begin{array}{c} \text{y}\\ 0 \end{array}\right]\right|\right|}_{2}^{2} \end{array}$$

where, **I** is the identity matrix and λ is the L2-norm regularization parameter (Hansen, 1992).

### 2.4. Apparent Inversion Efficiency

RF field inhomogeneities can affect the efficiency of the adiabatic inversion yielding an inversion angle $\theta_{inv}\neq 180^{{}^{\circ}}$ and reducing the factor 2 in Equation (1) to (1 − cosθ_*inv*_). In addition, dynamic processes such as magnetization transfer (MT) and cross-relaxation between immobile (bound) water associated with macromolecules and mobile (free) tissue water can produce a similar effect, further reducing the factor 2 in Equation (1) (Roscher et al., 1996; Gochberg et al., 1997; Does et al., 1998). Following the convention from Labadie et al. (2014), we can combine these processes together using the apparent inversion efficiency η defined as a percentage:

(4) $$\begin{array}{l} S_{\eta }\left(b,TI,TR\right)=\int_{0}^{\infty }\int_{0}^{\infty }(1-2\frac{\eta }{100}e^{-TI\cdot R_{1}}\\ \text{ +}e^{-TR\cdot R_{1}})e^{-b\cdot \bar{D}}p(R_{1},\bar{D})dR_{1}d\bar{D} \end{array}$$

We propose a two-step process to fitting the IR-IDE data using Equation (4). First, we solve the constrained linear problem assuming perfect inversion (Equation 3) to derive an approximate solution. This step can be implemented using non-negative least-squares optimization, and the regularization parameter can be optimized with various methods (Canales-Rodríguez et al., 2021). However, since in this step we seek merely an approximate solution, it is not necessary to optimize the value of λ for each voxel. The approximate solution is then used as a starting point in solving the nonlinear optimization problem:

(5) $$\begin{array}{l} \left[\hat{\text{p}};\hat{\eta }\right]=\underset{\text{p}>0,\eta }{\text{argmin}}{\left||{\text{M}}_{\eta }\text{p}-\text{y}|\right|}_{2}^{2} \end{array}$$

where, the elements of the encoding matrix **M**_η_, are calculated as the mean signal attenuations in the i-th experiment, *S*_η_(*b*_*i*_, *TI*_*i*_, *TR*_*i*_), evaluated over the 2D box function determined by the intervals [*R*_1,*j*_, *R*_1,*j*+1_] and $\left[{\bar{D}}_{j},{\bar{D}}_{j+1}\right]$ corresponding to the j-th spectral bin in the piecewise continuous approximation of $p\left(R_{1},\bar{D}\right)$. This second step of the spectral reconstruction is implemented using constrained non-linear optimization. The final solution, $\left[\hat{\text{p}};\hat{\eta }\right]$ yields estimates for both the T1-MD spectral components and the parameter η.

From multiple measurements with different combinations of joint IR and IDE weightings, **y**, we can reconstruct the T1-MD spectrum, $\hat{\text{p}}$, in each voxel. These piecewise continuous spectra can approximate arbitrary probability density functions of T1-MD values that may occur in healthy and pathological tissues.

### 2.5. Numerical Simulation and Phantom Experiments

We performed Monte Carlo (MC) experiments to validate the numerical stability of the T1-MD spectral reconstruction and to empirically optimize an experimental protocol suitable to run on a clinical scanner. Considering the range of T1- and diffusion-encoding parameters achievable on a clinical scanner and the time constraints for scanning human subjects, we generated a protocol for acquiring 304 IR-IDE DWIs comprising all combinations of 19 T1-weightings, i.e., (TI,TR) pairs, and 16 diffusion weightings, i.e., *b*-values. We conducted MC experiments assuming η = 90% and using mixtures of normal or lognormal T1-MD distributions (Remin et al., 1989) as our ground-truth spectra. For each distribution, we computed the 304 IR-IDE ground-truth signal attenuations and we generated multiple instances of noisy data with signal-to-noise ratio (SNR) levels similar to those achievable in the human brain. From 500 independent instances of simulated noisy measurements, we reconstructed the corresponding T1-MD spectra, quantified the means and standard deviations in all spectral bins, and compared the results to the original ground-truth distributions. We also investigated the effect of the reconstruction grid size on the numerical stability of the spectral estimation and its ability to distinguish multiple peaks.

Next, we tested the imaging capabilities of our IR-IDE pulse sequence and assessed the accuracy of our T1-MD measurement (experimental design, contrast encoding, signal representation, and numerical estimation) in quantifying the physical properties of a well-calibrated homogeneous spherical diffusion MRI phantom that mimics the average relaxation properties of brain tissues. The phantom was constructed using a 40% polyvinylpyrrolidone (PVP) polymer solution (Pierpaoli et al., 2009; Wagner et al., 2017; Sarlls et al., 2018) with nominal diffusivity and T1 of 0.53 μ*m*^2^/*ms* and 0.71 s, respectively, at room temperature. Diffusion within the phantom is expected to be uniform, isotropic and homogeneous, with a single mean diffusivity component and no signs of microscopic anisotropy or orientation dispersion. The homogeneity of the phantom at the macroscopic and microscopic scales allows us to concurrently assess potential sources of imaging artifacts such as ghosting, ringing, eddy current distortions, as well as potential errors in T1 and diffusion encodings due to source such as B1 inhomogeneities, gradient nonlinearities, concomitant fields, or eddy currents. We conducted an IR-IDE experiment in the phantom with the same protocol and reconstruction pipeline used for the *in vivo* exams. Finally, we compared the 2D T1-MD correlation spectroscopy images in the phantom to the nominal T1 and MD values.

### 2.6. Data Acquisition in Healthy Volunteers

We scanned three healthy volunteers using the IR-IDE protocol developed empirically based on the numerical simulation experiments. All subjects provided written and informed consent to participate in the study in accordance with a clinical protocol approved by the institutional review board (IRB) of the Intramural Research Program of the National Institute of Neurological Disorders and Stroke (NINDS). The MRIs were acquired on a conventional 3T Siemens Prisma scanner equipped with a 32-channel head RF coil and a gradient system that can achieve a maximum gradient strength of 80 mT/m/axis. We acquired 304 IR-IDE DWIs in 19 separate scans with different T1-weightings: one scan with no-IR and TR = 12 s, and 18 scans with TI values between 0.05 and 5 s and TR values between 1.63 and 10 s. For each scan, we first played out three discarded acquisitions (3 TRs) to establish the steady-state longitudinal magnetization followed by 16 consecutive acquisitions during the following TRs with a range of IDE weightings b = 50–3,600 *s*/*mm*^2^. All volumes were acquired with single-shot multislice EPI readout with TE = 98 ms, a field-of-view (FOV) of 220 mm on an 88 × 88 imaging matrix, a GRAPPA factor of 2, no partial Fourier sampling, 2.5 mm in-plane resolution, 20 axial slices with a 5 mm slice thickness grouped in two concatenations. The non-cubic voxel shape 2.5 × 2.5 × 5.0 mm was chosen to mitigate imaging artifacts due to Gibbs ringing while maintaining a high voxel SNR. The total scan time of the IR-IDE protocol was 51 min. All volunteers were instructed to minimize motion during the entire duration of the experiment. Additional scans were also acquired: a 30-direction DTI scan with b = 1,100 *s*/*mm*^2^, and the same EPI acquisition parameters as the IR-IDE scans; a 1-mm sagittal MP-RAGE (TE/TI/TR = 3.3/1,100/2,530 ms); and a 1-mm T2-weighted (T2W) Turbo-spin echo image (TE/TR = 72/8,000 ms) to serve as an anatomical reference for EPI distortion correction.

We processed all diffusion MRI data sets using the TORTOISE software package to correct for EPI distortions due to eddy currents and magnetic field inhomogeneities (Pierpaoli et al., 2010) and to register the DWIs to the corresponding T2W structural reference scan. We estimated tissue SNR levels by dividing the baseline signals (no IR, b = 0.05 *ms*/μ*m*^2^) averaged in tissue-specific regions-of-interest by the signal standard deviation measured in a noise region outside the brain. From the corrected IR-DWIs we computed voxelwise T1-MD spectra using 144 spectral components defined by bins with 12 MD values logarithmically spaced between 0.3 and 3.0 μ*m*^2^/*ms*, and 12 T1 values logarithmically spaced between 0.25 and 3.3 *s*. We repeated the analysis using different grid sizes with logarithmic spacing in order to better visualize signal components in specific spectral bands. We derived marginal distributions of subvoxel T1 values and MDs by integrating the T1-MD spectra along each dimension and compared the variability of the estimated spectra across subjects. Furthermore, based on visual inspection of whole-brain T1-MD distributions we manually selected spectral components that are consistent with different tissue types and integrated over the corresponding spectral bins to determine the signal fractions of these components in all subjects.

## 3. Results

### 3.1. Monte Carlo Simulations and Phantom Experiments

**Figure 2:**
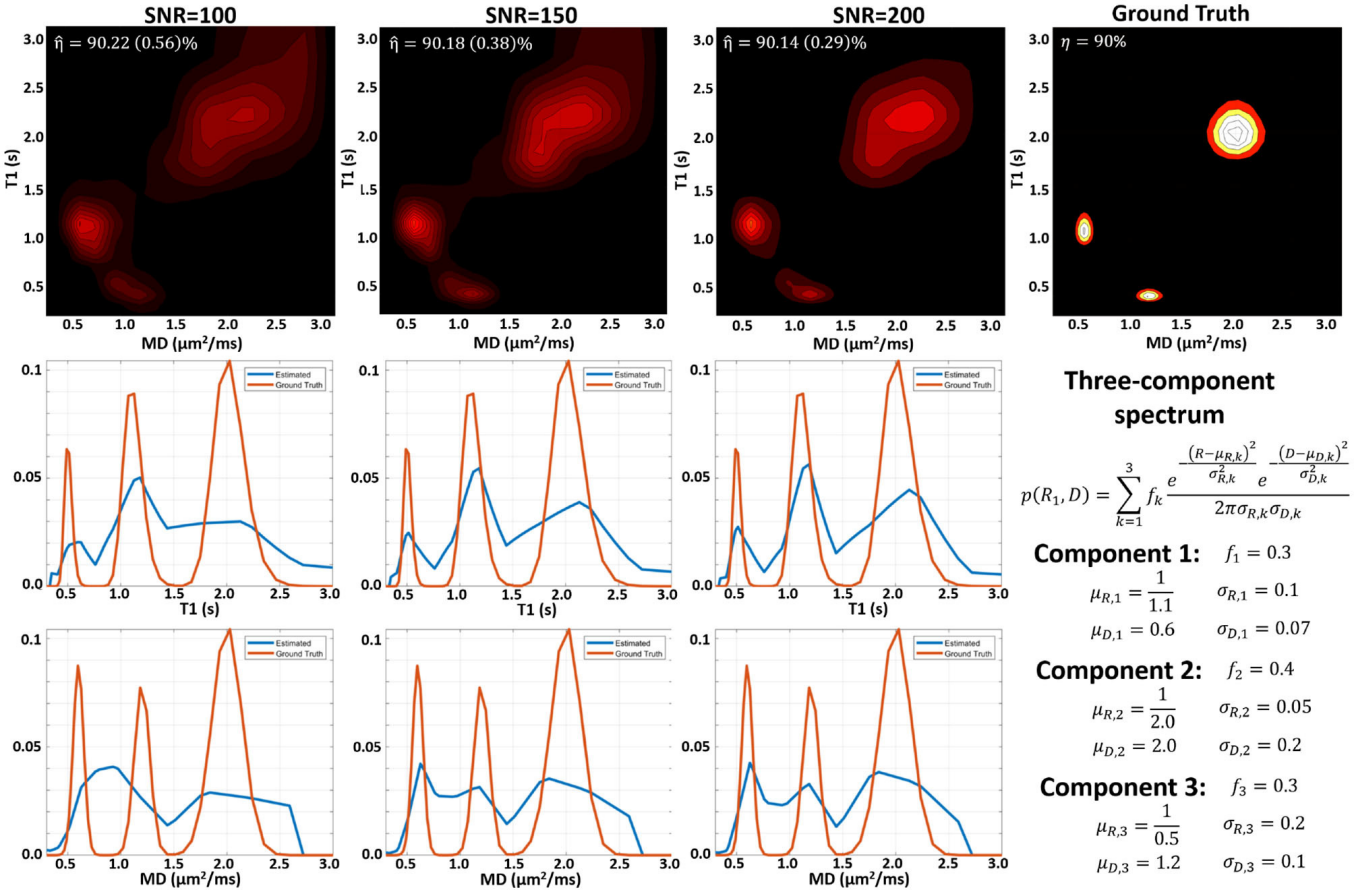
Monte Carlo experiments illustrating the dependence of the spectral reconstruction on measurement noise using the proposed protocol with 304 IR-IDE DWIs for a three-component T1-MD distribution. **(Top)** Comparison of ground-truth normalized spectrum (right), and the mean normalized reconstructed spectra for measurements with different SNR levels simulated using the IR-IDE protocol for scanning healthy volunteers. **(Middle)** Estimated T1 marginal distributions (blue line) derived from the T1-MD spectra in the top row compared to the ground-truth T1 marginal distribution (red line). **(Bottom)** Estimated mean diffusivity marginal distributions derived from the T1-MD spectra in the top row compared to the ground-truth MD marginal distributions.

MC simulation results suggest that we can estimate subvoxel multicomponent T1-MD spectra from a sufficiently large number of IR-DWIs with SNR levels that can be achieved on clinical MRI scanners. Figures showing examples of MC simulations using our clinical protocol and ground-truth spectra with one, two, and three components similar to those observed *in vivo* at different SNR levels are included as Supplementary Material. Figure 2 shows MC simulation results for a ground-truth spectrum containing a mixture of three components. The apparent inversion efficiency can be measured with very good precision and accuracy. The peak locations and relative signal fractions in the mean estimated and ground-truth correlation spectra and in the corresponding 1D marginal distributions are generally in good agreement. At higher SNR levels, the uncertainties in estimating the peak locations and amplitudes are lower, reducing the variance of the reconstructed spectra and the blurring in the estimated mean spectrum.

**Figure 3:**
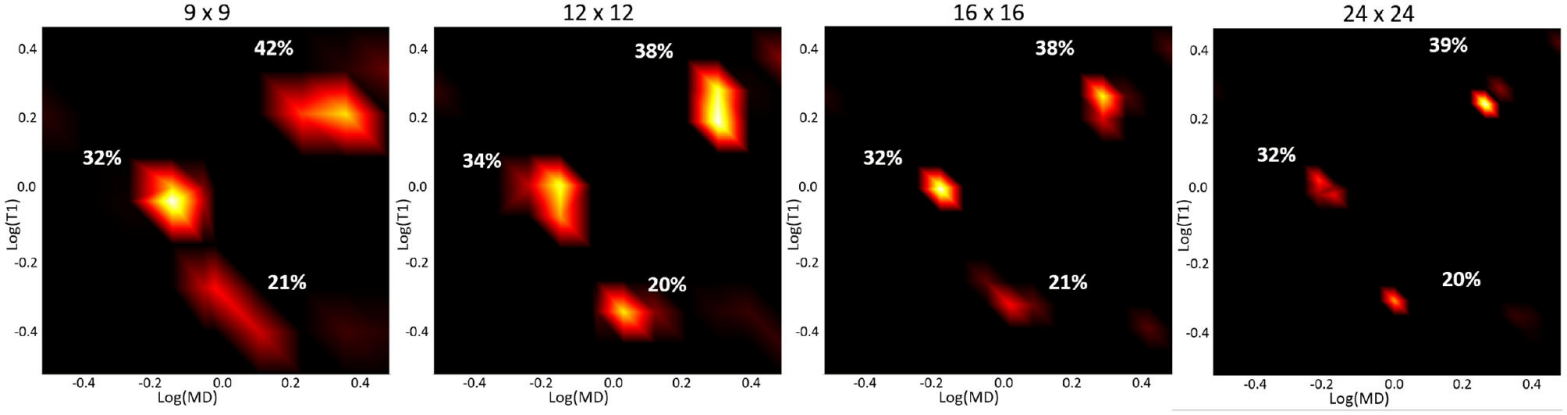
Log-log plots of T1-MD spectra reconstructed using different grid sizes from a noisy simulated data set (with the same T1-MD encodings as our clinical protocol) of the three-component ground-truth spectrum in Figure 2, assuming an SNR of 150. The peak locations and corresponding signal fractions (i.e., areas under the peaks) of each component, quantified as percentages of the total signal, are consistent across reconstructions using different grid sizes.

Apart from noise, the accuracy of the spectral reconstruction in our MC simulations is also affected by the finite number of measurements and the use of constraints in solving the poorly-conditioned problem. The constraints in Equation (5) make it difficult to establish analytical relations between the spectral resolution, accuracy, or precision and factors such as measurement noise or encoding parameters (b, TI, and TR) that apply generally to any ground-truth distribution. Nevertheless, the performance of the spectral reconstruction is relatively unaffected by our choice of the reconstruction grid (Figure 3). Because the estimated normalized spectra are piecewise continuous probability density functions (i.e., 2D box functions), the reconstruction grid (i.e., spectral bins) does not affect the characteristics of the solution. While in this study we used an empirical approach based on the MC experiments to derive a protocol suitable for use on a clinical scanner, the experimental design may be improved in the future with advanced sampling methods and optimization algorithms.

**Figure 4:**
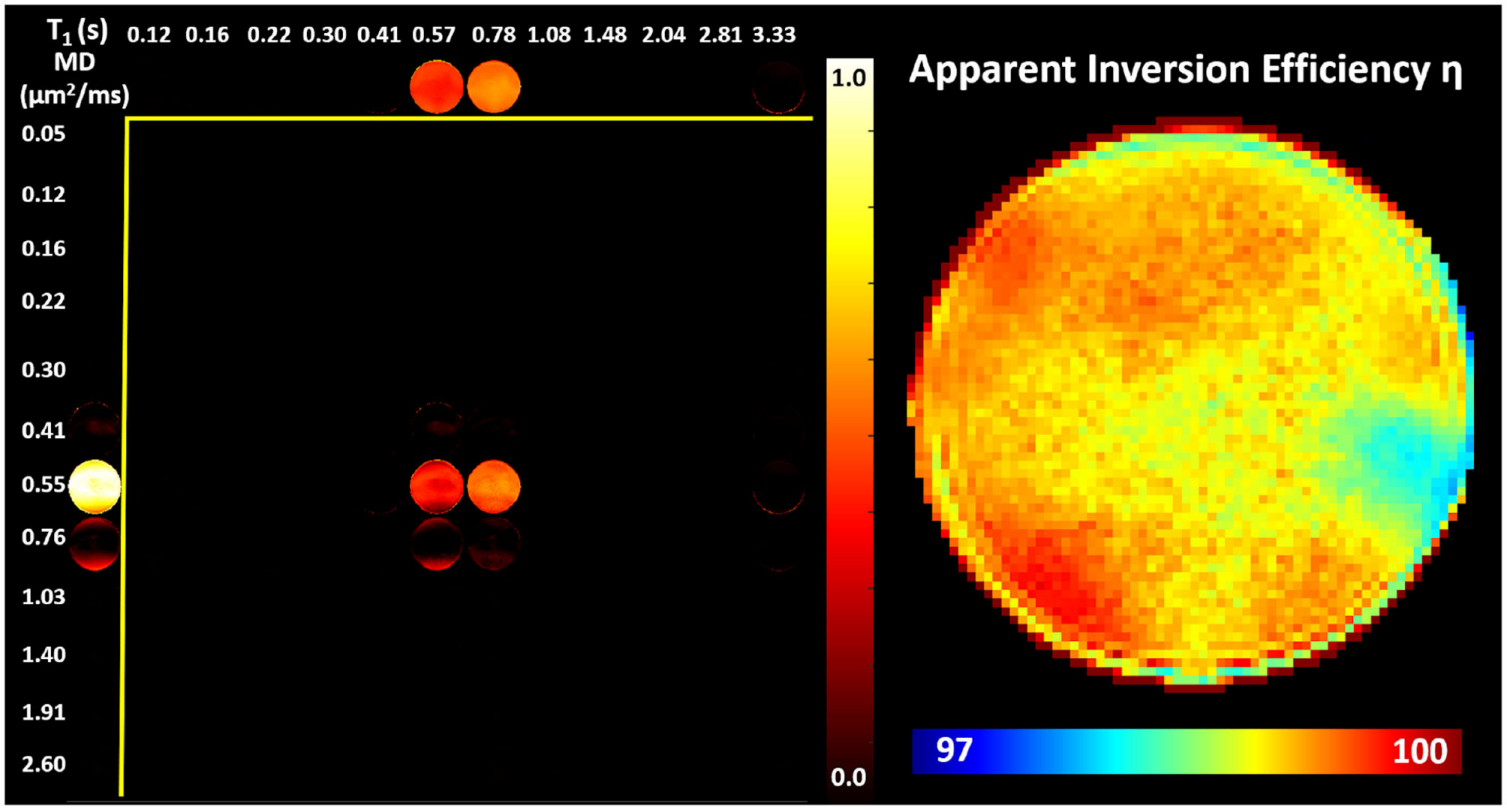
**(Left)** Maps of normalized T1-MD spectra measured in a well-calibrated MRI diffusion phantom with 40% PVP polymer concentration. **(Right)** The estimated inversion efficiency parameter η. Throughout the phantom, a single spectral peak can be observed centered around the nominal T1 and MD values for the phantom at room temperature 0.71*s* and 0.53μ*m*^2^/*ms*, respectively. The relatively low level of spatial inhomogeneities in individual spectral components supports the ability to accurately estimate the locations of T1-MD spectral peaks across large fields-of-view.

Images obtained in the PVP phantom showed single-peak T1-MD spectra throughout the phantom (Figure 4), with *MD* ≈ 0.55μ*m*^2^/*ms* and *T*1 ≈ 0.70*s*, in good agreement with the nominal values (Pierpaoli et al., 2009; Wagner et al., 2017; Sarlls et al., 2018). These results illustrate the ability of our imaging method to accurately encode and map T1-MD properties across large fields-of-view. The accuracy in estimating the MD of the PVP phantom confirms that the diffusion gradient amplitudes are scaled accurately to obtain the desired *b*-values. Meanwhile, the relatively small apparent inversion efficiency values suggest that RF inversion pulses are accurately calibrated and the T1-encoding in our sequence is not significantly affected by B1 inhomogeneities. Slight variations in the spatial-spectral uniformity of the estimated spectra may be caused by imaging distortions and unaccounted diffusion encoding contributions from magnetic field inhomogeneities, eddy currents, concomitant fields, or gradient non-linearities.

### 3.2. *In vivo* T1-MD Correlation Spectroscopic MRI

**Figure 5:**
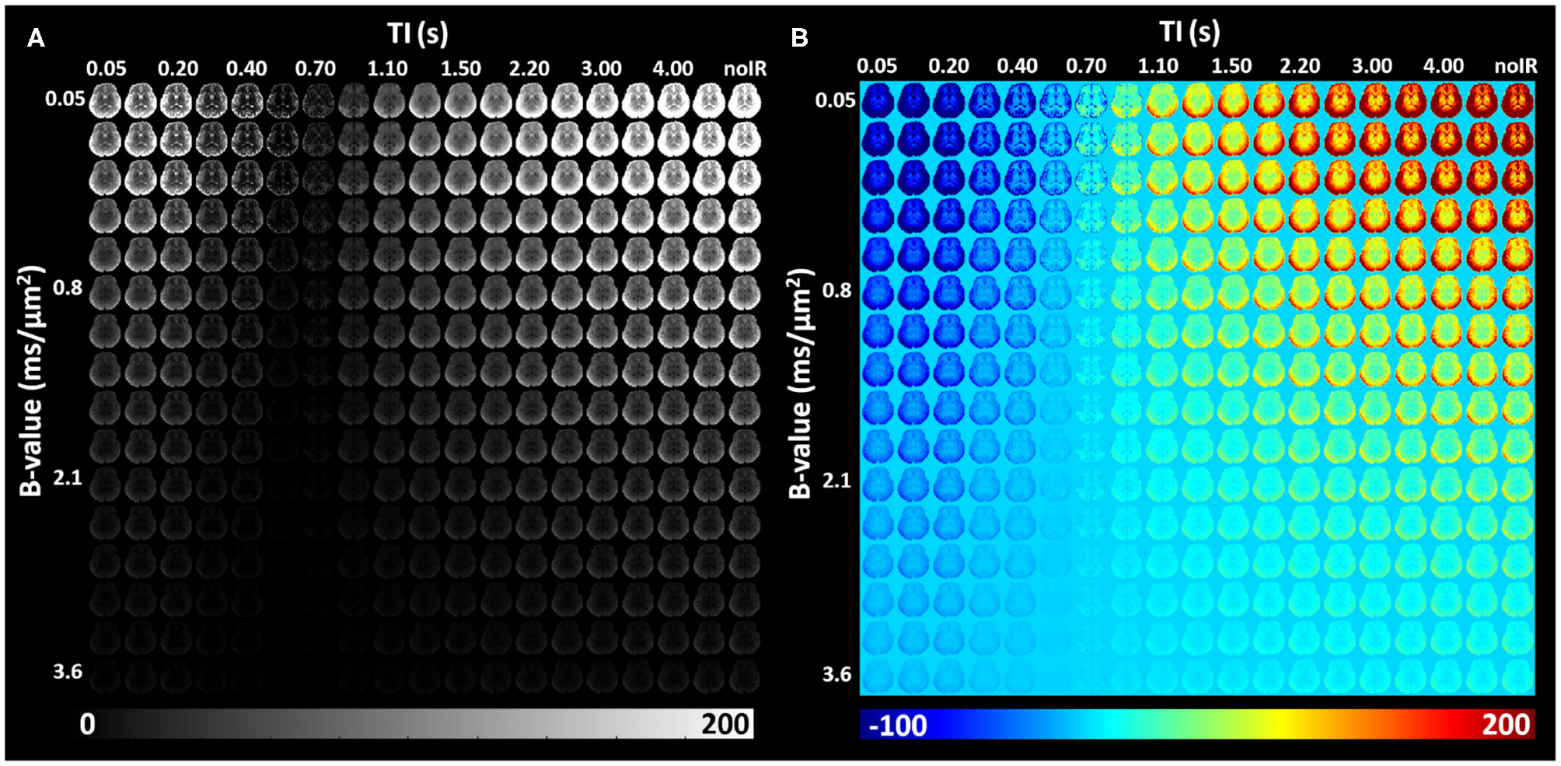
*In vivo* IR-IDE DWIs in a representative slice from a healthy volunteer: magnitude **(A)** and polarity-corrected **(B)** images.

IR-IDE DWIs showed good SNR in all study participants. Typical SNR values computed from non-attenuated signals were 250 in gray matter (GM), 125 in WM, 105 in the basal ganglia (BG), and 370 in cerebrospinal fluid (CSF). The relatively high SNR in GM is likely due to partial volume contributions from CSF, while the use of parallel imaging may also bias these tissue SNR estimates. Throughout the brain, signal attenuations due to diffusion and T1 contrasts showed a large dynamic range which was further increased after phase-sensitive IR correction (Figure 5).

**Figure 6:**
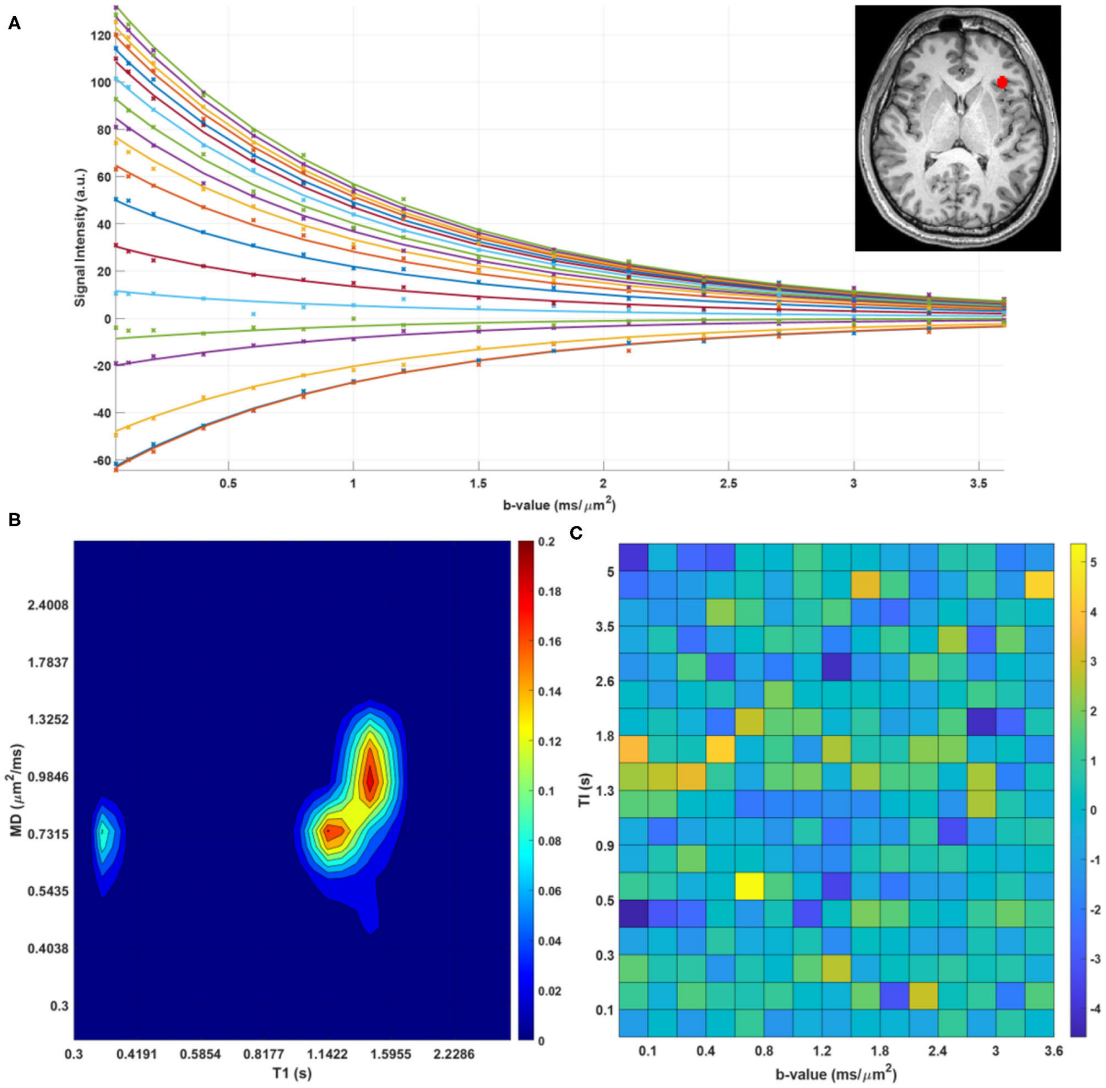
**(A)** Measured data points (stars) and estimated fit (solid lines) for a single voxel in the human brain. The different colors of lines/points correspond to different T1-encodings (i.e., separate scans). **(B)** Log-log contour plot of the corresponding normalized T1-MD correlation spectrum. **(C)** Corresponding residuals as a function of T1- and MD-weightings.

**Figure 7:**
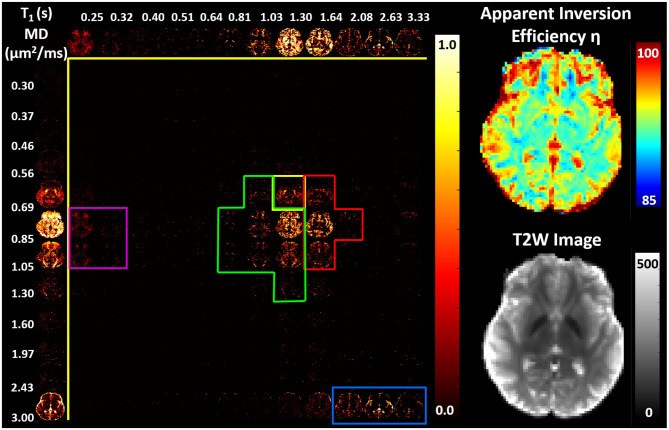
**(Left)** Maps of 2D probability density functions (i.e., 2D normalized spectra) of subvoxel T1-MD values reconstructed on a 12 × 12 grid, along with the corresponding marginal probability density functions (i.e., 1D normalized spectra) of subvoxel T1 values (top row) and subvoxel MD values (left column) derived from the 304 phase-corrected IR-IDE MRIs in Figure 4. **(Right)** Corresponding maps of apparent inversion efficiency η (top) and the non-attenuated signal, i.e., T2-weighted image (bottom), in the same slice. Spectral components specific to WM (green), GM (red), basal ganglia (yellow), short-T1 WM (magenta), and CSF (blue) were delineated manually based on visual inspection of whole-brain T1-MD spectra.

We found predominantly T1-MD spectra with one, two, or three distinct components in the brains of healthy volunteers. Figure 6 shows the raw data points and reconstructed normalized T1-MD correlation spectrum in a representative brain tissue voxel along with the residuals to the spectral reconstruction as a function of the encoding parameters. The independence of the residuals on the T1 and diffusion encoding suggests that the spectral representation explains the data well. *In vivo* estimates of the inversion efficiency parameter, η, showed values of ~ 89% in WM, 95% in GM, and 98% in CSF (Figure 7), in good agreement with an earlier study (Labadie et al., 2014). Maps of normalized T1-MD correlation spectra and corresponding 1D marginal distributions revealed signal components that were spatially consistent with specific tissue types such as WM, GM, BG, and CSF (Figure 7).

The CSF signal showed a single peak (Figure 7, blue contour) with long T1 and large MD values (Kwong et al., 1991) that was well-isolated from parenchymal spectral components. Due to the relatively coarse spatial resolution, many voxels in the cortex and around the ventricles showed both CSF and parenchymal components. These partial volume contributions could be promptly separated in the reconstructed spectra. Even though the CSF peak can be easily distinguished in both spectral dimensions, it has a narrower range of MD values centered around ≈ 3.0μ*m*^2^/*ms*. The larger range of the CSF T1 values 2.0–3.3 s may reflect partial volume variations across measurements with different TIs due to subject/physiological motions or the inflow of fresh blood (i.e., non-inverted spins) from neighboring slices especially in GM (Trampel et al., 2004) during the T1 encoding duration. Diffusion encoding on the other hand occurs during a fixed short duration of the evolution of transverse magnetization and is localized to the excited slice.

**Figure 8:**
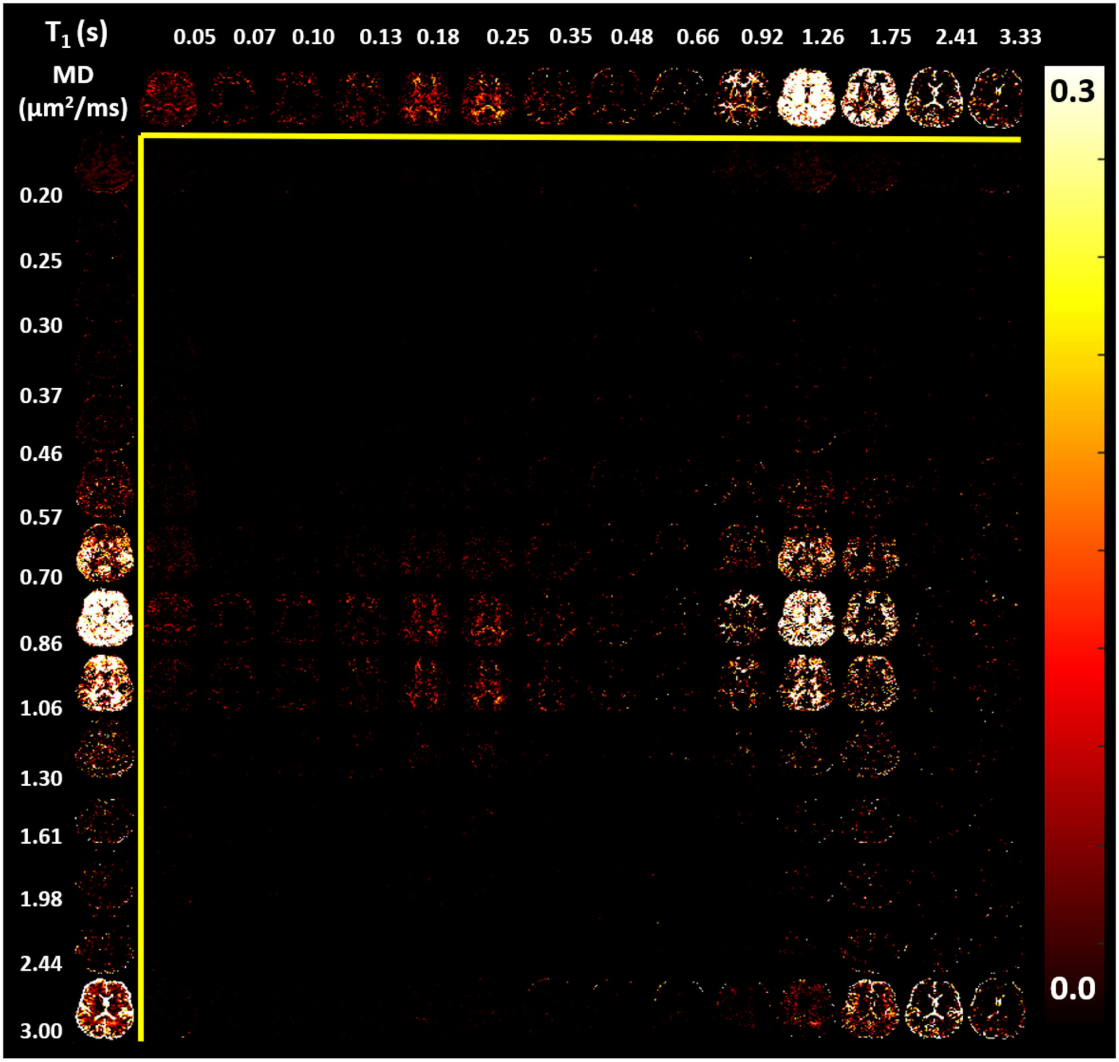
Maps of estimated normalized T1-MD spectra (same slice shown in Figure 7) reconstructed using a 14 × 14 grid with bin locations chosen using logarithmic spacing to better visualize the short-T1 spectral component in WM: 14 MD values with logarithmic spacing between 0.2 and 3.0 μ*m*^2^/*ms*; and 14 *T*_1_ values with logarithmic spacing between 0.05 and 3.33 s. (Top row) The corresponding marginal distribution of T1 values. (Left column) The corresponding marginal distribution of MD values. The short-T1 peak contains components in the range of 130–350 *ms*.

In the parenchyma, WM showed the largest heterogeneity of subvoxel T1-MD properties, revealing two distinct peaks. The major peak (Figure 7, green outline) was relatively broad with intermediate values of T1 1.0–1.3 s and MD 0.85–1.00 μ*m*^2^/*ms*. The second peak with shorter T1 values ≈ 250 ms was specific to the major WM pathways and accounted for ≈ 10.2% of the WM signal (Figure 7, magenta outline). It may reflect processes associated with myelin water as suggested in previous *in vivo* studies (Labadie et al., 2014). The MD properties of the short-T1 peak were similar to those of other parenchymal components suggesting that this peak may be an indirect measure of myelin water via MT or chemical exchange (Avram et al., 2010). The short-T1 WM component could be better visualized using a higher resolution reconstruction grid in Figure 8. The reconstructed spectra in WM, and more generally in the brain parenchyma, did not reveal very low MD components that may suggest the presence of a very restricted diffusion compartment (i.e., dot compartment; Tax et al., 2020).

In GM we observed a single component, with significantly longer T1 values 1.30–2.08 *s* and slightly lower MD values compared to WM (Figure 7, red contour). The lowest parenchymal MD values 0.56–0.69 μ*m*^2^/*ms* were specific to the BG, including the putamen and caudate nucleus (Figure 7, yellow contour), in agreement with previous *in vivo* studies (Avram et al., 2019). The localization of low MD components in the BG is evident in the marginal MD distribution components (Figure 7, left column). The BG also contained spectral components found in other tissues.

**Figure 9:**
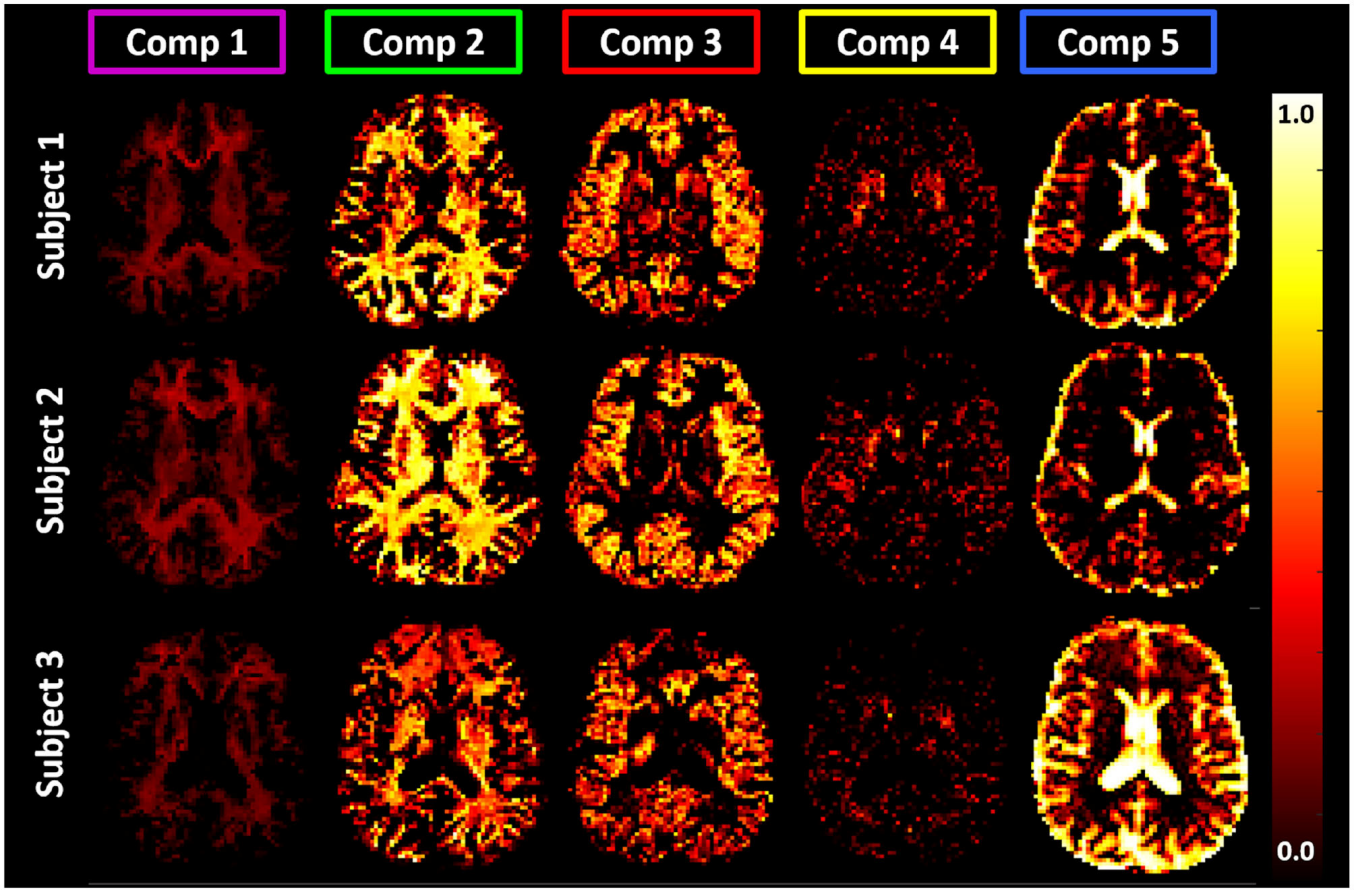
Maps of signal fractions corresponding to the principal T1-MD spectral components delineated with color-coded boundaries in Figure 7: Component 1 (magenta): short-T1 WM with T1 and MD values in the range of 0.0–0.32 *s* and 0.69–1.05 μ*m*^2^/*ms*; Component 2 (green): WM with T1 and MD values in the range of 0.64–1.30 *s* and 0.56–1.30 μ*m*^2^/*ms*, respectively; Component 3 (red): GM with T1 and MD values in the range of 1.30–2.08 *s* and 0.56–1.05 μ*m*^2^/*ms*, respectively; Component 4 (yellow): BG with T1 and MD values in the range of 1.03–1.30 *s* and 0.56–0.69 μ*m*^2^/*ms*, respectively; and Component 5 (blue): CSF with T1 and MD values in the range of 1.64–3.33 *s* and 2.43–3.00 μ*m*^2^/*ms*, respectively. Matching axial slices in three healthy volunteers show similar anatomical features corresponding to these spectral domains.

The one-dimensional (1D) marginal distributions derived from the T1-MD correlation spectra were in very good agreement with results from other T1 (Labadie et al., 2014) and MD (Avram et al., 2019) relaxation spectroscopic MRI studies, further supporting the accuracy of the T1 and MD preparations and the spectral reconstruction. The signal fractions obtained by integrating the manually selected spectral regions-of-interest (ROIs) corresponding to 1 CSF and 4 parenchymal components were consistent across the three study participants (Figure 9) and showed slight improvements in anatomical specificity compared to the 1D marginal distributions (Figures 7, 8). The *ad hoc* approach for defining the spectral ROI components in Figure 7 will be improved and automated in future studies with larger cohorts of subjects. While these preliminary findings did not reveal any remarkable T1-MD correlations in the healthy brain, they encourage further studies with larger cohorts of healthy volunteers and patients to assess the reproducibility and establish the clinical applicability and utility of T1-MD CS-MRI.

The spatial-spectral characteristics of T1-MD distributions did not depend on the locations or sizes of the spectral bins in the reconstruction (Figures 7, 8), as expected from our numerical simulation results (Figure 3). Estimating T1-MD spectra with different reconstruction grids can improve visualization of specific spectral bands. For example, Figure 8 reveals that the T1 values of the short-T1 component in WM are in the range of 0.13–0.35 *s*. In general, reconstructions using denser grids require longer processing times and can be more sensitive to measurement noise as they aim to estimate more unknowns (i.e., spectral components). In addition, reconstructions using dense grids distribute the spatial-spectral information over a large number of spectral components maps, making it more difficult to identify spatially coherent anatomical features. Future studies will use numerical simulations to optimize not only the reconstruction grids but also the experimental protocols (TI-b weightings) for specific clinical applications based on high-quality T1-MD CS-MRI data sets measured in larger cohorts of healthy volunteers and patients.

## 4. Discussion

Mapping the subvoxel heterogeneity of magnetic relaxation and diffusion parameters, e.g., T2 (Mackay et al., 1994), T1 (Labadie et al., 2014), MD (Avram et al., 2019) using a clinical MRI scanner may yield new clinical biomarkers. Recent human studies have demonstrated the ability to acquire multidimensional CS-MRI data (Tax et al., 2017; Kim et al., 2018; Slator et al., 2019). In this study, we integrate two separate spectroscopic MRI methods with 1D encodings (Labadie et al., 2014; Avram et al., 2019) to image, for the first time, correlation spectra of T1 and MDs in the human brain. There are several considerations for our choice. Firstly, clinical studies have repeatedly demonstrated the sensitivity of T1 (e.g., FLAIR) and diffusion (e.g., mADC) contrasts to various pathophysiological tissue processes such as perturbations in cellular metabolism, microstructural remodeling, iron accumulation, changes in protein and lipid concentrations. Secondly, preclinical experiments suggest that water diffusivity in tissues correlates less with T1 than with T2 (Benjamini and Basser, 2017). Furthermore, because T1 and diffusion preparations modulate the longitudinal and transverse magnetizations, respectively, we can independently control these contrasts to achieve a wide range of T1-MD weightings. Finally, IR-preparation provides twice the signal dynamic range with excellent sensitivity for short and long TI preparations.

The novel IR-IDE sequence (Figure 1) overcomes several challenges to efficiently acquire whole-brain MRIs with a wide range of T1-MD weightings. Such measurements are traditionally very time-consuming due to the long T1-preparation durations, and the need to account for diffusion anisotropy. To accelerate T1-encoding we interleaved inversion and acquisition modules (Park et al., 1985; Oh et al., 1991; Listerud et al., 1996) across multiple slices and adjusted both TI and TR concurrently. T1-encoding efficiency may be further improved by sampling multiple time points during the IR recovery (Look and Locker, 1970; Lee, 2000; Shah et al., 2001; Steinhoff et al., 2001; Hutter et al., 2018), by using MR fingerprinting (McGivney et al., 2018; Tang et al., 2018; Kim et al., 2019), or compressed sensing (Bai et al., 2015). Meanwhile, the diffusion signal in tissues is modulated by the exact timings, amplitudes, and orientations of the diffusion-sensitizing gradient waveforms. Using very economical gradient sampling schemes (Avram et al., 2018) we can derive whole-brain mADC-weighted MRIs (Jones et al., 1999) with arbitrary b-value very efficiently from only a few DWIs per b-value. Nevertheless, in heterogeneous tissues, the mADC-weighted signal decay does not capture the diffusion-diffusion correlations required to encode diffusion isotropically in each microscopic water pool (Westin et al., 2016; Topgaard, 2017; Avram et al., 2019). On the other hand, the IDE preparation (Avram et al., 2019) weighs all diffusion processes isotropically in a single excitation, removing both macroscopic and microscopic effects of diffusion anisotropy and drastically accelerating the scan.

The IDE signal can be viewed as a practical, one-dimensional simplification of a general representation that breaks down the voxel signal into contributions from a distribution of diffusion tensors whose diffusion ellipsoids have various sizes, shapes, and orientations and correspond to slowly-exchanging microscopic water pools (Westin et al., 2016; Topgaard, 2017; Avram et al., 2019; Magdoom et al., 2021). Therefore, it is naturally suited for relaxation correlation spectroscopic analysis, such as T1-MD CS-MRI. The signal representation in Equation (4) can account for very general distributions that may arise in healthy and diseased tissues.

Previous CS-MRI studies with human subjects (Labadie et al., 2014; Kim et al., 2018) have used spatial regularization to enforce smoothness of the reconstructed spectra across neighboring voxels. In this study, we did not employ any non-local (i.e., spatial) constraints in the voxel-wise T1-MD spectral reconstruction to maintain the sensitivity to subtle local differences in tissue components, which is important in diagnostic applications. The multidimensional spectral reconstruction may be improved by using other techniques such as Monte Carlo reconstruction (Prange and Song, 2009), compressed sensing (Bai et al., 2015), marginal distribution constrained (MADCO) reconstruction (Benjamini and Basser, 2016), Bayesian estimation (McGivney et al., 2018), or deep learning (Pirkl et al., 2020).

Sources of error in MD-encoding include undesired diffusion weighting contributions due to concomitant field gradients, eddy currents, gradient non-linearities, and B0 field inhomogeneities (Avram et al., 2019). Meanwhile, the quantitation and interpretation of T1 spectra can be affected by: the adiabatic inversion efficiency which depends on spatial variations in the RF field (B1 inhomogeneities); dynamic tissue-specific processes such as MT and cross-relaxation between bound (macromolecular) water and free tissue water, as well as the water exchange across different T1 components; physiological effects such as the inflow of fresh blood (Trampel et al., 2004) into the imaging slice; and partial volume instabilities due to tissue motions during the long duration of the experiment.

High-SNR and large dynamic range are intrinsic requirements for disentangling multiexponential decays in RS-MRI (Figure 2). The SNR can be improved on clinical scanners by increasing the voxel size, for instance. However, in high-SNR MRIs, signal-dependent imaging artifacts (e.g., Gibbs ringing, aliasing) can be considerably larger than the noise level. Furthermore, in long-duration scans with human subjects, additional factors, such as physiological motions, blood flow, tissue perfusion, or active metabolic processes may cause partial volume instabilities that fluctuate above the noise level, potentially compounding the quantitation bias.

RF field inhomogeneities typically produce spatially smooth variations in the inversion efficiency. However, inversion efficiency maps measured *in vivo* (Figure 7) showed a notable tissue dependence (lower values in WM compared to GM and CSF), suggesting that the longitudinal magnetization available at excitation may also reflect tissue-specific processes such as magnetization transfer, cross-relaxation, or chemical exchange. This tissue dependence, first reported in an *in vivo* T1 RS-MRI study (Labadie et al., 2014), was attributed to differences in MT and cross-relaxation properties among brain tissues (Labadie et al., 2014). The authors of that study incorporated contributions from RF inhomogeneities and tissue-specific processes into a general parameter called apparent inversion efficiency, η. This pragmatic empirical description allows the estimation of T1 spectra *in vivo* but assumes a uniform inversion efficiency in all subvoxel signal components.

In general, the interpretation of any T1 measurement should carefully consider the effects of dynamic processes like MT which depend on the pattern of RF pulses during a multi-slice scan. In IR experiments, these processes can lead to signal changes that are difficult to disentangle from fast longitudinal relaxation and affect primarily the quantitation of short-T1 components (Edzes and Samulski, 1978; Gochberg et al., 1997; Does et al., 1998), such as those observed in WM (Labadie et al., 2014). In our multi-slice IR experiment, an additional confound may arise from the TI dependence of MT effects. When a given slice is inverted using an on-resonance slice-selective IR-pulse, the neighboring slices can experience the same IR pulse as off-resonance saturations compounding their MT effects. Since between the inversion and excitation pulses of each slice, the number of out-of-slice inversions changes with TI, measurements with different TIs could reflect different amounts of MT saturation. The contributions of MT in IR experiments have been characterized for simple two-pool (bound and free water) systems (Gochberg and Gore, 2003, 2007). Extending these frameworks to a spectrum of T1 tissue components requires many additional unknowns to account for the coupling between spectral components and different measurements that are not feasible for an *in vivo* study like ours.

Due to the relatively long TE needed to accommodate the IDE preparation, the T1-MD spectra are strongly T2-weighted and may differ from similar measurements using shorter TEs. The TE = 98 ms is significantly longer than the T2 of myelin water (<35 ms) resulting in an almost complete attenuation due to the transverse relaxation of the direct myelin water contribution to the measured signal. Consequently, the short-T1 peak in Figures 7, 8 may not quantify myelin water directly, but likely reflects processes such as MT and chemical exchange between myelin water and water in other tissue compartments. Further decreasing the TE in images with T1 and diffusion encoding may be possible by using high-performance gradient systems (Jones et al., 2018), stimulated echo sequences, and center-out readout trajectories (Avram et al., 2014).

## 5. Conclusions

Building on the established radiological utility and sensitivity to pathophysiology of DWI and FLAIR images, this study takes significant steps toward a non-parametric quantitation of the subvoxel heterogeneity of two important biophysical tissue properties. It lays out the methodology for measuring whole-brain IR-IDE MRIs (pulse sequence and experimental design) and illustrates, as a proof-of-principle, how from those measurements one can map T1-MD spectra (signal representation and spectral reconstruction). Mapping the subvoxel landscape of joint T1-diffusion properties may reveal sensitive tissue signal components for diagnosing and characterizing neuroinflammatory and neurodegenerative diseases, traumatic brain injury, ischemic stroke, or cancer.

## Data Availability Statement

The raw data supporting the conclusions of this article will be made available by the authors, without undue reservation.

## Ethics Statement

The studies involving human participants were reviewed and approved by The Institutional Review Board (IRB) of the Intramural Research Program (IRP) of the National Institute of Neurological Disorders and Stroke (NINDS) within the National Institutes of Health (NIH). The patients/participants provided their written informed consent to participate in this study.

## Author Contributions

AA: conceptualization, methodology, software, investigation, data curation, writing—original draft, writing—review and editing, visualization, supervision, and project administration. JS: methodology, investigation, resources, and writing—review and editing. PB: conceptualization, methodology, resources, writing—review and editing, and funding acquisition. All authors contributed to the article and approved the submitted version.

## Conflict of Interest

The authors declare that the research was conducted in the absence of any commercial or financial relationships that could be construed as a potential conflict of interest.
