# Supplementary material for: Whole-Brain Imaging of Subvoxel T1-Diffusion Correlation Spectra in Human Subjects
Source: Front Neurosci. 2021 Jun 11;15:671465. doi: 10.3389/fnins.2021.671465 (PMC8232058; doi:10.3389/fnins.2021.671465)
Supplement: Supplementary file 1 [file Data_Sheet_1.PDF]

Supplementary Material

1 SUPPLEMENTARY FIGURES

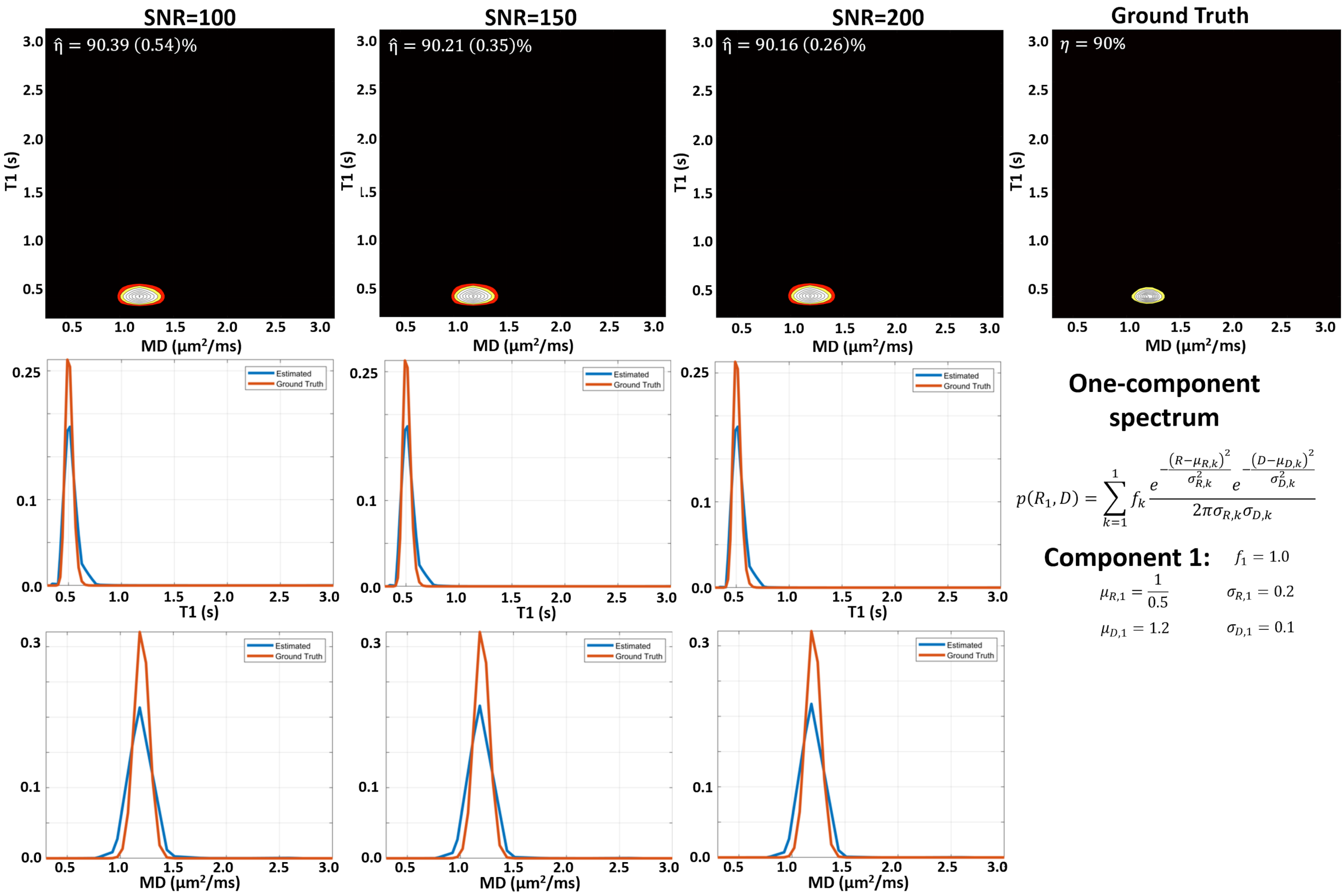

$$p(R_1, D) = \sum_{k=1}^{1} f_k \frac{e^{-\frac{(R-\mu_{R,k})^2}{\sigma_{R,k}^2}} e^{-\frac{(D-\mu_{D,k})^2}{\sigma_{D,k}^2}}}{2\pi\sigma_{R,k}\sigma_{D,k}}$$

$f_1 = 1.0$, $\mu_{R,1} = \frac{1}{0.5}$, $\sigma_{R,1} = 0.2$, $\mu_{D,1} = 1.2$, $\sigma_{D,1} = 0.1$

Figure S1. Monte Carlo experiments illustrating the dependence of the spectral reconstruction on measurement noise using the proposed protocol with 304 IR-IDE DWIs for a single component T1-MD distribution. **Top row**: Comparison of ground truth normalized spectrum (right), and the mean normalized reconstructed spectra for measurements with different SNR levels simulated using the IR-IDE protocol for scanning healthy volunteers. **Middle row**: Estimated T1 marginal distributions (blue line) derived from the T1-MD spectra in the top row compared to the ground truth T1 marginal distribution (red line). **Bottom row**: Estimated mean diffusivity marginal distributions derived from the T1-MD spectra in the top row compared to the ground truth MD marginal distributions.

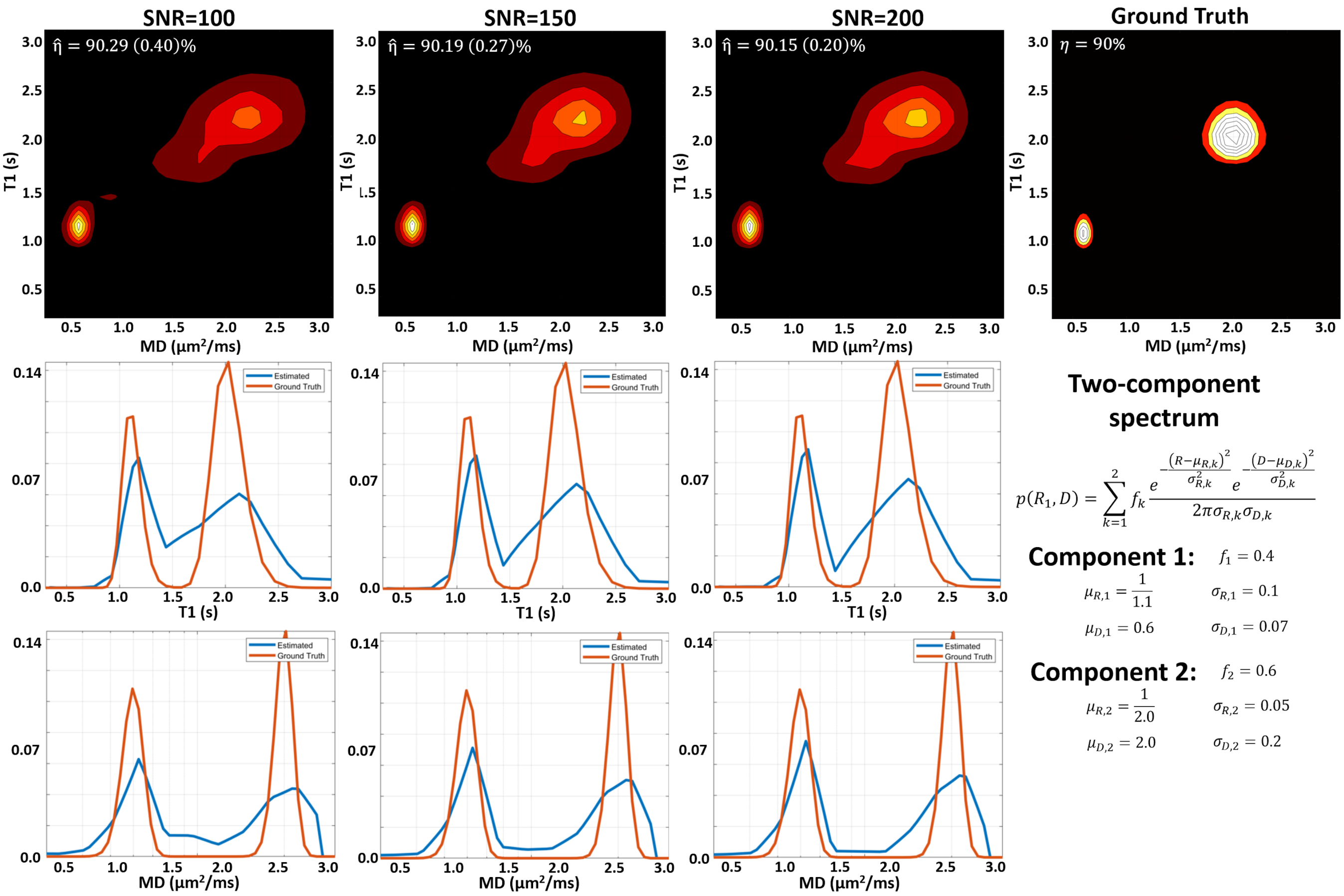

Figure S2. Monte Carlo experiments illustrating the dependence of the spectral reconstruction on measurement noise using the proposed protocol with 304 IR-IDE DWIs for a two-component T1-MD distribution. **Top row**: Comparison of ground truth normalized spectrum (right), and the mean normalized reconstructed spectra for measurements with different SNR levels simulated using the IR-IDE protocol for scanning healthy volunteers. **Middle row**: Estimated T1 marginal distributions (blue line) derived from the T1-MD spectra in the top row compared to the ground truth T1 marginal distribution (red line). **Bottom row**: Estimated mean diffusivity marginal distributions derived from the T1-MD spectra in the top row compared to the ground truth MD marginal distributions.

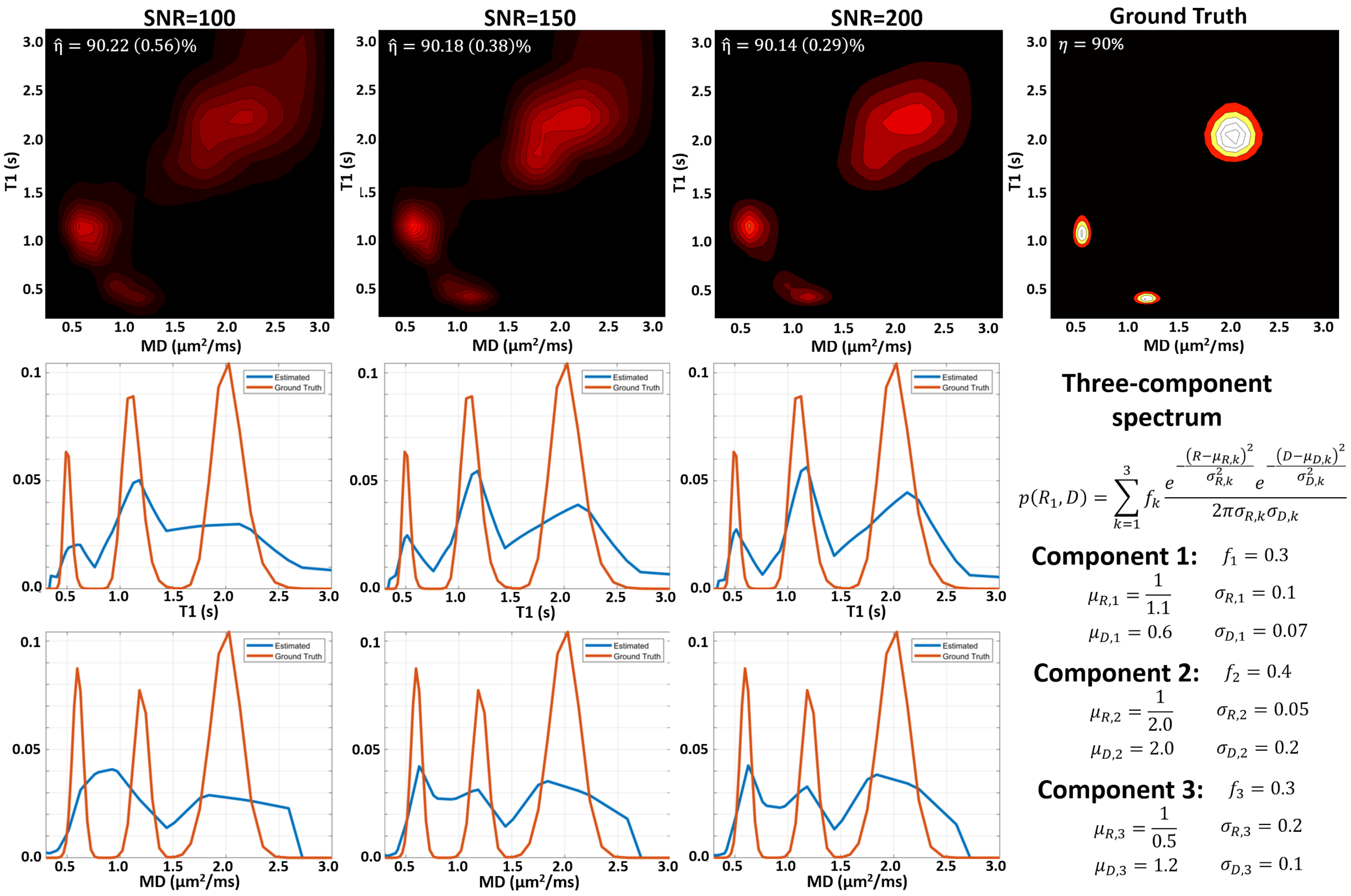

Figure S3. Monte Carlo experiments illustrating the dependence of the spectral reconstruction on measurement noise using the proposed protocol with 304 IR-IDE DWIs for a three-component T1-MD distribution. **Top row**: Comparison of ground truth normalized spectrum (right), and the mean normalized reconstructed spectra for measurements with different SNR levels simulated using the IR-IDE protocol for scanning healthy volunteers. **Middle row**: Estimated T1 marginal distributions (blue line) derived from the T1-MD spectra in the top row compared to the ground truth T1 marginal distribution (red line). **Bottom row**: Estimated mean diffusivity marginal distributions derived from the T1-MD spectra in the top row compared to the ground truth MD marginal distributions.
